# Somatic Experiencing® for patients with low back pain and comorbid posttraumatic stress symptoms – a randomised controlled trial

**DOI:** 10.1080/20008198.2020.1797306

**Published:** 2020-08-18

**Authors:** Tonny Elmose Andersen, Hanne Ellegaard, Berit Schiøttz-Christensen, Anna Mejldal, Claus Manniche

**Affiliations:** a Department of Psychology, University of Southern Denmark, Odense, Denmark; b Spine Centre of Southern Denmark, University of Southern Denmark, Odense, Denmark; c Department of Clinical Research, University of Southern Denmark, Odense, Denmark

**Keywords:** Post-traumatic Stress, Somatic Experiencing®, pain, low back pain, RCT, Estrés postraumático, Experiencia Somática®, dolor, lumbalgia, ensayo clínico aleatorizado, 创伤后应激, 体感疗法, 疼痛, 腰痛, RCT, • The current study is the first randomized controlled trial evaluating the effect of a full 12-session program of Somatic Experiencing (SE) for comorbid PTSS and low back pain.• SE + physiotherapeutic intervention was compared to the physiotherapeutic intervention alone. No significant group differences were found on any of the outcomes at any timepoints.• Both groups achieved a large significant reduction in disability (20%) at 6 and 12-months follow-up.• Also, both groups achieved a small reduction in PTSS.

## Abstract

**Background:**

Low back pain (LBP) and comorbid post-traumatic stress symptoms (PTSS) are common after traumatic injuries, and a high level of PTSS is associated with more severe pain and pain-related disability. Few randomised controlled trials (RCT) exist targeting comorbid PTSS and chronic pain, and only one has assessed the effect of Somatic Experiencing®.

**Objective:**

The aim of this study was to assess the effect of Somatic Experiencing® (up to 12 sessions) + physiotherapeutic intervention (4–8 sessions) (SE+PT) compared with the physiotherapeutic intervention alone (4–8 sessions) (PT) for pain-related disability in LBP with comorbid PTSS.

**Methods:**

The study was a two-group RCT in which participants (*n* = 114) were recruited consecutively from a large Danish Spine Centre. Patients were randomly allocated to either SE+PT or PT alone. Outcomes were collected at baseline before randomisation, 6 and 12-month post-randomisation. The primary outcome was pain-related disability as measured with the modified version of the Roland Morris Disability Questionnaire at 6-month post-randomisation. Secondary outcomes were PTSS, pain intensity, pain-catastrophising, kinesiophobia, anxiety and depression.

**Results:**

No significant group differences were found on any of the outcomes at any timepoints. Both groups achieved a significant reduction in pain-related disability (20–27%) as measured by the Roland Morris Disability Questionnaire at 6 and 12-month follow up. Also, both groups achieved a small reduction in PTSS.

**Conclusions:**

Although significant effects were achieved for both groups, the additional SE intervention did not result in any additional benefits in any of the outcomes.

## Abstract (es)

**Antecedentes:** La lumbalgia y los síntomas comórbidos de estrés postraumático (SCET) son comunes luego de lesiones traumáticas, y un alto nivel de los SCET está asociado con dolor más severo y con discapacidad asociada al dolor. Existen escasos ensayos clínicos aleatorizados enfocados en los SCET y en dolor crónico, y solo uno ha evaluado el efecto de la Experiencia Somática®.

**Objetivo:** El objetivo de este estudio fue el de evaluar el efecto de la Experiencia Somática® (hasta un máximo de 12 sesiones) adicionada a la intervención fisioterapéutica (entre 4 a 8 sesiones) (ES+IF), comparada con la intervención fisioterapéutica sola (entre 4 a 8 sesiones) (IF), sobre la discapacidad asociada al dolor en lumbalgia con SCET.

**Métodos:** El estudio consistió en un ensayo clínico aleatorizado de dos grupos para el que se reclutó a participantes (*n*=144) consecutivamente de un gran Centro Danés de Columna Vertebral. Los pacientes fueron distribuidos aleatoriamente al grupo de ES+IF o al grupo de solo IF. Los puntos de corte se realizaron de base antes de la aleatorización, y a los 6 y 12 meses luego de la aleatorización. El resultado principal era la discapacidad asociada a dolor, medida mediante la versión modificada del Cuestionario de Discapacidad de Roland Morris a los seis meses luego de la aleatorización. Los resultados secundarios fueron los SCET, la intensidad del dolor, la catastrofización sobre el dolor, la quinesofobia, la ansiedad, y la depresión.

**Resultados:** No se encontraron diferencias significativas entre los grupos sobre los resultados medidos, en ningún punto de corte. Ambos grupos alcanzaron una reducción significativa de la discapacidad asociada a dolor (20 – 27%), medida mediante el Cuestionario de Discapacidad de Roland Morris a los 6 y a los 12 meses. Además, ambos grupos alcanzaron una reducción pequeña en los SCET.

**Conclusiones:** A pesar de que se alcanzaron resultados significativos en ambos grupos, la intervención adicional mediante Experiencia Somática® no aportó ningún beneficio adicional sobre ninguno de los resultados.

## Abstract (zh)

**背景**:腰痛 (LBP) 和并发创伤后应激症状 (PTSS) 在创伤后很常见, 且高水平的PTSS与更严重的疼痛和疼痛相关性残疾相关。很少有针对PTSS与慢性疼痛共病的随机对照试验 (RCT), 并且只有一项评估了体感疗法的效果。

**目的**:本研究旨在评估体感疗法 (多达12个疗程) +理疗干预 (4-8个疗程) (SE + PT) 相较于单纯理疗干预 (4-8个疗程) (PT) 在并发PTSS的LBP中对疼痛相关性残疾的影响。

**方法**:本研究有两组RCT, 从一个大型丹麦脊柱中心连续招募参与者 (*n* = 114) 。患者被随机分配到SE + PT组或PT组中。在随机分组前基线, 随机分组后6个月和12个月时收集结果。主要结果为随机分配后6个月时使用修订版《罗兰·莫里斯残疾问卷》测量的疼痛相关性残疾。次要结果为PTSS, 疼痛强度, 疼痛灾难化, 运动恐惧, 焦虑和抑郁。

**结果**:在任何时间点, 任何结果均未发现显著组间差异。在6个月和12个月的随访时, 两组患者均实现了疼痛相关性残疾 (由《罗兰·莫里斯残疾问卷》测量) 的显著降低 (20-27％) 。而且, 两组的PTSS均有小幅降低。

**结论**:尽管两组均取得了显著效果, 额外的SE干预并未给任何结果带来任何额外益处。

While it is well known that neck pain is common after motor vehicle collisions (MVC), it is less well known that low back pain (LBP) is equally as common as neck pain, with a prevalence of 37% (Bortsov et al., 2014; Cassidy, Carroll, Côté, Berglund, & Nygren, 2003). Also, psychological distress, such as post-traumatic stress symptoms (PTSS), is common after MVCs and traumatic injuries, and numerous studies have found comorbid PTSS to be associated with more severe pain and pain-related disability (Andersen, Andersen, & Andersen, 2014; Andersen, Karstoft, Brink, & Elklit, 2016; Moeller-Bertram, Keltner, & Strigo, 2012). Unfortunately, few randomised controlled trials (RCT) exist addressing comorbid PTSS and chronic pain.

Whereas most evidence-based interventions for the treatment of PTSD are cognitive-behavioural therapies (CBT) (Watkins, Sprag, & Rothbaum, 2018), more body-oriented approaches like Somatic Experiencing® (SE) (Levine, 2010) are emerging. SE differs from CBT interventions by uniquely focusing on interoception and musculoskeletal sensations. SE does not rely on verbal cognitive processing of the traumatic memories. The rationale behind SE is to help the patient to access the so-called body memory of the traumatic event and teach the patient to monitor arousal and downregulate it by staying in the present moment with attention to both unpleasant and pleasant sensations. In that sense, SE resembles mindfulness in facilitating sustained attention to interoceptive sensations in the present moment and, at the same time, creating room for new interoceptive experiences that contradict the negative sensations associated with the trauma (Levine, 2010; Payne, Levine, & Crane-Godreau, 2015). Hence, traumatic memories are processed by a bodily-oriented focus on self-regulation of arousal.

To our knowledge only two RCTs of SE exist (Andersen, Lahav, Ellegaard, & Manniche, 2017; Brom et al., 2017) and only the study by Andersen et al. (2017) was with a sample of patients with chronic LBP. In general, very few RCTs have addressed comorbid PTSS in the context of pain (Andersen et al., 2017; Beck, Coffey, Foy, Keane, & Blanchard, 2009; Dunne, Kenardy, & Sterling, 2012).

In a sample of patients with LBP and comorbid PTSS (N = 91), Andersen et al. (2017) assessed the effect of a brief SE intervention (6–10 sessions) in addition to treatment-as-usual (supervised exercises for low back pain) on PTSS. At the 12-month follow up, the group that received the additional SE intervention experienced significantly lower levels of PTSS compared with treatment-as-usual alone. The result corresponded to a large effect size. However, there were no group differences in reduction of pain or pain-related disability. The study by Brom et al. (2017) investigated the effect of SE (15 sessions) in a pain-free sample with PTSS (N = 63). At the 15-week follow up, the SE group had achieved a significant reduction in PTSS compared with the waitlist group, a result that also corresponded to a large effect size.

Beck et al. (2009) assessed the effect of group cognitive behavioural therapy (CBT) for comorbid PTSS and pain in survivors of serious MVCs compared with a minimal contact condition (*n* = 44). Only the CBT group achieved a large significant reduction in PTSS. However, none of the groups experienced significant reductions in pain. The final trial by Dunne et al. (2012) was a pilot study (*n* = 26) on whiplash-associated disorders investigating the effect of trauma-focused CBT compared with a waitlist condition. In contrast to Andersen et al. (2017) and Beck et al. (2009), a moderate reduction was found in pain, pain-related disability and PTSS compared with the control group (Dunne et al., 2012). However, the results should be interpreted with caution, given the small sample size and the lack of an active control condition. Moreover, the effects were small and not above the minimal clinically important difference (MCID = the smallest change in a treatment outcome that an individual patient would identify as important). Only change in PTSS was considered to be above the MCID (Dunne et al., 2012).

It is still debated whether PTSS and pain are simply co-occuring or mutually maintaining conditions (Otis, Keane, & Kerns, 2003; Ravn, Hartvigsen, Hansen, Sterling, & Andersen, 2018). A number of mutually maintaining mechanisms have been suggested (for a review of the theoretical frameworks and empirical studies, see Otis et al., 2003 and Ravn, Hartvigsen, et al., 2018). Factors such as catastrophising, hyperarousal and avoidance behaviours may maintain and exacerbate both pain and PTSS. Also, when pain and PTSS are tied to the same event, pain symptoms may serve as a reminder of the traumatic event and thereby lead to re-experiencing symptoms (Ravn, Eskildsen, Johnsen, Sterling, & Andersen, 2020). Hence, targeting these potentially mutually maintaining mechanisms with an additional psychotherapeutic intervention designed to treat PTSS may also have a positive impact on pain related disability and distress (Asmundson & Katz, 2009; Sharp & Harvey, 2001). Also, interventions targeting pain-related avoidance may have a positive effect on PTSS. For instance, Robinson, Theodore, Dansie, Wilson, and Turk (2013) found that exposure therapy targeting pain-related fear-avoidance behaviours in whiplash-injured patients significantly reduced both PTSS and pain-related disability. Recently, Sullivan et al. (2017) found that an intervention designed to reduce catastrophising following work-related injury was effective in reducing both disability and PTSS. The results are theoretically in accordance with the mutual maintenance model (Sharp & Harvey, 2001) and Andersen et al.’s (2016) finding that the association between pain and PTSS was mediated by pain-related fear-avoidance beliefs and pain-catastrophising.

Hence, the aim of the current study was to assess whether an additional SE intervention, in combination with a physiotherapeutic intervention for comorbid PTSS and LBP after accident and injury-related trauma would reduce pain-related disability. First, we hypothesised that an additional SE intervention (SE+PT) would reduce pain-related disability compared with the physiotherapeutic intervention alone (PT) at the 6-month follow up. Secondly, compared with the PT alone, we hypothesised that the additional SE intervention (SE+PT) would reduce all secondary outcome scores at the 6-month follow up (PTSS, pain, pain-catastrophising, kinesiophobia, anxiety, and depression).

## 1. Methods

### 1.1. Study design and participants

The study was a two-group RCT in which participants (*n* = 114) were recruited consecutively from a large Danish spine centre in the Region of Southern Denmark, between January 2016 and December 2017. Patients with LBP were included in the study if they were between 18 and 70 years of age and had experienced a traumatic event (MVC or injury) within the last 5 years (DSM-IV criteria A: APA, 1994) and screened positive for PTSS (See section ‘secondary outcomes’). Exclusion criteria were known psychiatric diseases and drug dependence or other ongoing psychotherapeutic interventions (see protocol: Andersen, Ellegaard, Schiøttz-Christensen, & Manniche, 2018).

Ethics approval was obtained from the local science ethics committee (trial number S-20,150,136) and all participants gave written informed consent before study entry. The study was funded by the Danish Victims Fund, which has been set up by the Danish Parliament, with the objective being to fund projects and activities that provide further knowledge of, or support for, victims or groups of victims of crimes and road accidents. The funding body has not played any role in the study design or the collection, analysis and interpretation of data.

**Figure 1.**
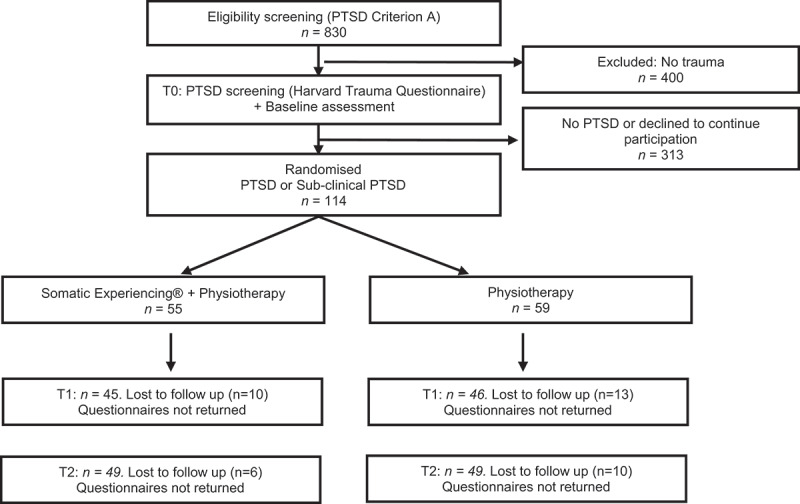
Flow diagram of patients.

Figure 1 illustrates the patient flow in this study.

T0 = baseline before randomisation, T1 = 6-month follow up, T2 = 12-month follow up.

### 1.2. Randomisation and masking

Patients were randomised using random permuted blocks of six. Randomisation procedures were administered by a project nurse, blinded to treatment assignment. Patients were randomly allocated to either: Somatic Experiencing® (SE) plus physiotherapeutic intervention (PT) or PT alone, and the interventions were initiated within 2 weeks after randomisation. Primary and secondary outcomes were collected at baseline before randomisation (T0), 6 (T1) and 12-month (T2) post-randomisation. The study statistician was blinded to treatment allocation and the patients were blinded to the hypotheses.

### 1.3. Interventions

Both intervention groups (SE+PT and PT) received the same physiotherapeutic intervention, as described below. However, one group received the additional SE intervention (SE+PT). In this group, both the PT intervention and the SE intervention were delivered as weekly sessions, most often within the same week but never on the same day. For pragmatic reasons, sessions were scheduled to best suit the patient; however, within the timeframe of 12–16 weeks in total.

### 1.4. Physiotherapeutic intervention

The PT intervention was an individualised functional treatment with the aim being to improve daily functioning. The intervention was based on guided exercises for LBP, and exposure to feared movements or exercises. The intervention did not apply any manual treatment, such as massage or manipulations of muscles and joints. The intervention was delivered in 4–8 weekly sessions, each approximately ½-1 hour, by physiotherapists in the centre and according to the European guidelines for the management of chronic LBP (Airaksinen et al., 2006).

### 1.5. The additional Somatic Experiencing® intervention

The SE intervention involved up to 12 sessions of one-hour SE therapy, delivered weekly by one of two certified SE therapists (a physiotherapist or a psychotherapist) with several years of experience in SE and pain management. The SE intervention was delivered according to the nine-step model as outlined by Peter Levine (Levine, 2010). The nine steps are intertwined processes starting with the facilitation of a safe therapeutic environment that supports a mindful exploration of bodily sensations. During the process, the patient is encouraged to experience how the body alternates between pleasant and unpleasant sensations. The intervention focus is on gradually eliciting awareness of body sensations associated with the traumatic event and encouraging the patient to access feelings and bodily sensations associated with the trauma. During therapy, the patient’s physical responses, such as breathing and bodily posture are addressed, and the patient is encouraged to shift between sensations associated with the trauma and bodily sensations that are experienced as a safe place or a source of strength and comfort as means to enhance self-regulation. An overview of the programme is outlined in Andersen et al. (2018).

### 1.6. Primary outcome

The primary outcome was pain-related disability as measured with the modified version of the Roland Morris Disability Questionnaire (RMDQ-23; Patrick et al., 1995) at 6-month post-randomisation. The RMDQ-23 measures the level of disability symptoms related to LBP on 23 statements covering six different domains: physical ability/activity, sleep/rest, psychosocial level of functioning, household management, eating, and pain frequency. Each statement is scored 1 if the patient feels that the statement is descriptive of their circumstances and scored 0 if not. Hence, the disability sum score ranges from 0 (no disability) to 23 (maximal disability). Scores are converted to percentages with 23 corresponding to 100% disability. Both internal consistency (α = 0.84 to 0.96) and test-retest reliability (r = 0.83 to 0.91) of the RMDQ are good (Smeets, Köke, Lin, Ferreira, & Demoulin, 2011). In the current study, internal consistency measured by Cronbach’s alpha was T0-T3 α = 0.82; 0.90; 0.91.

### 1.7. Secondary outcomes

PTSS were measured with the Harvard Trauma Questionnaire, Part IV [HTQ: Mollica, Caspi-Yavin, Bollini, & Truong, 1992). PTSS were calculated as the total of 16 items, each scored on a 4-point Likert scale (0 = not at all to 3 = very often). The 16 items relate to the PTSD symptom clusters: avoidance (7 items), re-experiencing (4 items), and hyperarousal (5 items). Patients were included if they scored above a predefined cut-off criterion of at least one re-experiencing item, two avoidance items, and one hyperarousal item (each item scored as ≥ 2). The HTQ is a self-report measure of PTSD that has previously been reported as having an 88% concordance with interview-based estimates of PTSD (Mollica et al., 1992). In the current study, internal consistency measured by Cronbach’s alpha was T0-T3 α = 0.72; 0.90; 0.92.

Traumatic exposure was assessed by the Life Event Checklist-5 (LEC-5: Weathers et al., 2013). The LEC was slightly modified by excluding events that are not so common in Denmark or relevant to the current context. Hence, ‘severe human suffering’ and ‘exposure to toxic substances’ were removed. Patients were asked to mark which of the 16 traumatic events they have either been directly exposed to or witnessed. Also, patients were asked to indicate which event they perceived as the primary traumatic event (index trauma).

Pain intensity was measured as the average of three numerical rating scales (pain NRS: Manniche et al., 1994) ranging from 0–10 (0 = no pain, 10 = worst imaginal pain). Patients were asked to rate their current pain intensity, peak pain intensity, and average pain intensity over the past 2 weeks. The scale is commonly used in LBP and has shown good psychometric properties (Manniche et al., 1994). In the current study, internal consistency measured by Cronbach’s alpha was T0-T3 α = 0.85; 0.92; 0.93.

Fear of re-injury due to movement was measured with the Tampa Scale for Kinesiophobia (TSK: Kori, Miller, & Todd, 1990). TSK is a 17-item self-report scale on which patients are asked to rate their level of agreement with each item on a 4-point Likert scale with the total score ranging from 17 to 68, with higher scores indicating higher levels of kinesiophobia. The scale is commonly used in diverse chronic pain samples and has good construct and predictive validity (Roelofs, Goubert, Peters, Vlaeyen, & Crombez, 2004; Vlaeyen, Kole-Snijders, Boeren, & van Eek, 1995). In the current study, internal consistency measured by Cronbach’s alpha was T0-T3 α = 0.67; 0.78; 0.77.

The Pain Catastrophising Scale (PCS: Sullivan, Bishop, & Pivik, 1995) was used to measure catastrophic thinking related to pain. The patients were asked to reflect on past painful experiences, and to indicate on a 5-point Likert scale (0 = not at all, 4 = all the time) the degree to which they experienced each of 13 thoughts or feelings when in pain. The higher the total score on the PCS, the higher the level of pain catastrophising. The scale has excellent psychometric properties and has been validated in both clinical and non-clinical samples (Kjøgx et al., 2014). In the current study, internal consistency measured by Cronbach’s alpha was (T0-T3 α = 0.88; 0.92; 0.95).

The Hospital Anxiety and Depression Scale (HADS: Zigmond & Snaith, 1983) was used to assess levels of anxiety and depression. The scale consists of two subscales each with seven items measuring symptoms of anxiety (HADS-A) and symptoms of depression (HADS-D). The score on each subscale ranges from 0–21. The scale is commonly used in somatic patients and is a well-validated questionnaire with good psychometric properties (Zigmond & Snaith, 1983). In the current study, internal consistency measured by Cronbach’s alpha was T0-T3 α = 0.81; 0.92; 0.91.

### 1.8. Statistical Analysis

Differences in baseline variables by group were tested using chi-square statistics for categorical variables and t-tests for continuous variables. The associations between primary and secondary outcomes and intervention were investigated using multilevel mixed-effects linear models (LMM) These models make it possible to deal efficiently with missing values due to dropout, assuming the dropout pattern is missing at random (MAR). Thus, all available data were used, and intention-to-treat analyses applied.

Fixed effects included time point and a group × time-point interaction term, where we additionally adjusted for sex and age. Because participants were randomised to either intervention or control group, the interaction tested for the existence of group-by-time-point interaction.

We included a random effect in the model for each subject, allowing each subject to have their individual intercept. For every outcome, the necessity of a random slope was tested and included in cases where it represented a significant improvement in the model (i.e., the trajectory of outcome could vary randomly per subject).

Effect sizes where reported as Cohens *d* as follows: small = 0.20, medium = 0.50, and large = 0.80. According to the literature, the minimal clinically important difference (MCID) in pain-related disability was determined to be a 30% reduction on the RMDQ compared with baseline (Jordan, Dunn, Lewis, & Croft, 2006; Lauridsen, Hartvigsen, Manniche, Korsholm, & Grunnet-Nilsson, 2006; Patrick et al., 1995). Lauridsen et al. (2006) estimated the MCID using a seven-point transition question and a NRS for importance. Responsiveness was operationalised using standardised response mean, area under the receiver operating characteristics curve, and cut-point analysis in primary and secondary care patients with LBP.

All statistical significance tests were two-tailed with α = 0.05. Analyses were conducted using STATA version 15 (StataCorp, College Station, Tx, USA) and SPSS version 26 (IBM Corp, Armonk, NY, USA).

## 2. Results

### 2.1. Sample characteristics

In total, 114 patients were included in the analysis. Of those, 66% were females. The mean age was 41 years (SD = 11.8). The mean number of years with chronic LBP was 3 years (SD = 3.74). The majority of patients had experienced multiple traumatic events; however, the most common index traumas reported were traffic accidents (47.8%), physical assaults (52.2%) and work-related accidents (39.3%).

The unadjusted means and SD for all outcomes and timepoints for both groups are shown in Table 1. At baseline, there were no significant group differences on any of the outcomes with the exception of a weak but significant difference between the two groups in PTSS (HTQ). However, since this was a randomised study, we must assume this difference to have occurred by chance. There were no significant differences (*p* > 0.05) between completers and non-completers in age or on any of the primary or secondary outcomes at baseline.

**Table 1.** Unadjusted means (SD) for outcomes by treatment group and time point.

	Baseline		6-month		12-month	
	*SE+Phys* *(n = 55)*	*Phys* *(*n* = 59)*	*SE+Phys* *(n = 45)*	*Phys* *(n = 46)*	*SE+Phys* *(*n* = 49)*	*Phys* *(n = 49)*
*Primary outcome*						
RMDQ (0–100)	64.22 (19.02)	68.90 (19.32)	47.00 (26.96)	53.67 (26.16)	51.11 (28.00)	50.17 (27.04)
*Secondary outcomes*						
HTQ (0–48)	23.81 (7.15)	26.28 (6.49)	21.35 (10.90)	23.12 (9.01)	21.20 (10.78)	21.52 (11.06)
NRS (0–10)	6.33 (2.11)	6.33 (1.80)	4.27 (2.54)	4.39 (2.07)	4.61 (2.51)	4.69 (2.52)
TSK (17–68)	45.65 (5.45)	46.16 (4.82)	41.15 (6.30)	42.81 (6.01)	41.56 (6.23)	42.05 (5.89)
PCS (0–52)	27.24 (8.47)	26.76 (8.69)	18.08 (10.64)	18.70 (9.42)	19.11 (12.11)	19.83 (10.77)
HADS-D (0–21)	7.51 (3.86)	7.83 (3.27)	7.44 (5.09)	6.55 (4.26)	6.77 (4.62)	6.69 (4.27)
HADS-A (0–21)	10.12 (3.15)	9.58 (3.77)	9.05 (4.76)	8.12 (4.26)	9.55 (4.57)	8.63 (4.12)

n = the number of participants with primary outcome data at each time point. RMDQ = Roland Morris Disability Questionnaire; HTQ = Harvard Trauma Questionnaire; NRS = Mean pain intensity on a Numerical Rating Scale; PCS = Pain Catastrophising Scale; HADS-D/A = Hospital Anxiety and Depression Scale;

SE = Somatic Experiencing®; Phys = Physiotherapy.

The mean number of SE sessions delivered was 10 (SD = 3) delivered within 12–16 weeks. The PT intervention was delivered within the same timeframe and all patients received between 6 and 8 sessions, each of ½-1 hour.

### 2.2. Treatment outcomes

Firstly, we investigated whether the outcomes changed over time, and secondly, whether the two treatment groups differed at any timepoint. The mixed-effects models of the group differences over time are shown in Table 2.

**Table 2.** Treatment effects expressed as predicted adjusted mean differences between SE+phys and phys at each time point.

	Baseline			6-month			12-month		
	*Mean Difference*	*95% CI*		*Mean Difference*	*95% CI*		*Mean Difference*	*95% CI*	
*Primary outcome*									
RMDQ (0–100)	−4.83	−11.51	1.58	−7.68	−16.74	1.38	−2.98	−14.06	8.09
*Secondary outcomes*									
HTQ (0–48)	−2.79*	−5.38	−0.20	−2.09	−5.53	1.35	−1.40	−5.97	3.18
NRS (0–10)	−0.01	−0.70	0.69	0.04	−0.87	0.94	−0.003	−0.99	0.99
TSK (17–68)	−0.45	−2.28	1.39	−2.22*	−4.42	−0.02	−1.23	−3.81	1.26
PCS (0–52)	0.87	−2.82	4.01	−0.10	−4.17	3.97	−0.27	−4.94	4.40
HADS-D (0–21)	−0.11	−1.33	1.11	0.51	−1.31	2.33	0.43	−1.31	2.18
HADS-A (0–21)	0.65	−0.66	1.96	0.67	−0.88	2.23	0.30	−1.61	2.20

RMDQ = Roland Morris Disability Questionnaire; HTQ = Harvard Trauma Questionnaire; NRS = Mean pain intensity on a

Numerical Rating Scale; PCS = Pain Catastrophising Scale; HADS-D/A = Hospital Anxiety and Depression Scale.

*P < 0.05. SE = Somatic Experiencing®; Phys = Physiotherapy (reference group).

There was a significant main effect of time on the primary outcome, pain-related disability, as measured by the RMDQ. Contrasts revealed that the decrease in RMDQ from baseline to the 6-month follow up (T1) (est. = −15.96, se = 2.386, *p <* 0.001) and from baseline to the 12-month follow up (T2) (est. = −14.7, se = 2.680, *p < *0.001) was significant. There was no difference detected between T1 and T2 (est. = 1.19, se = 2.596).

Likewise, significant improvements from baseline to T1 were found on the secondary outcomes: PTSS (HTQ), pain intensity (NRS), pain-catastrophising (PCS), kinesiophobia (TSK), and anxiety (HADS-A), again with no differences from T1 to T2. There was no improvement measured for depression (HADS-D) from baseline to T1 (est. = −0.74, se = 0.388) nor from T1 to T2 (est. = 0.13, se = 0.420). Regarding anxiety, although the patients improved significantly from baseline to T1 (est. = −1.22, se = 0.373, *p < *.01), this improvement diminished, with no significant difference from baseline to T2 (est. = −0.75, se = 0.465). Together, these trajectories demonstrate that any improvement occurred in the time from baseline to first follow up (T1), with a stagnation between the first and second follow up (T2).

### 2.3. Treatment outcomes by group

Investigating the interaction between timepoint and group (SE+PT and PT) for each outcome, we found that specifically, for pain-related disability (RMDQ), the difference between the two groups was sizable, though non-significant (est. = −7.78, se = 4.624), with the SE+PT group having the lowest score, however this difference diminished by T2 (est. = −2.98, se = 5.649). No significant differences between groups were found for any of the secondary outcomes at any timepoints, besides kinesiophoia (TSK), where a slight significant difference was found at T1 (est. = −2.220, se = 1.124) with the SE+PT group scoring lower than the PT group. However, this difference decreased at T2 and became non-significant.

**Figure 2.**
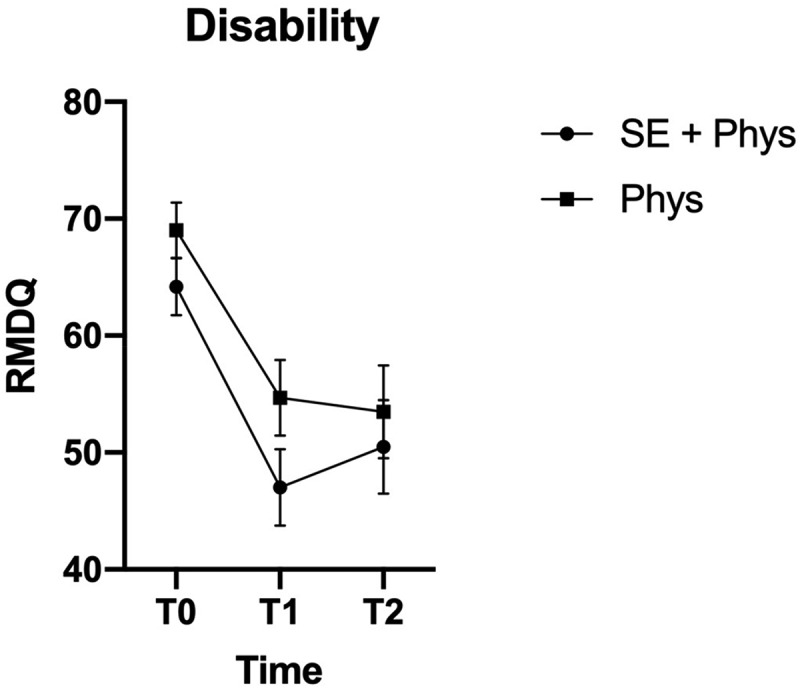
Marginal means and confidence intervals for pain-related disability by treatment group and time point.

Thus, the analysis revealed no significant interaction between group and timepoint on the main outcome and only one weaker interaction on a secondary outcome, namely kinesiophobia (TSK) at the first follow up. Figures 1 and 2 show the change in pain-related disability (RMDQ) and PTSS (HTQ) at all timepoints for the two groups.

Marginal means and 95% confidence intervals on the RMDQ (Roland morris disability questionnaire). T0 = baseline before randomisation, T1 = 6-month follow up, T2 = 12-month follow up. SE+Phys = Somatic Experiencing® + Physiotherapy, Phys = physiotherapy.

**Figure 3.**
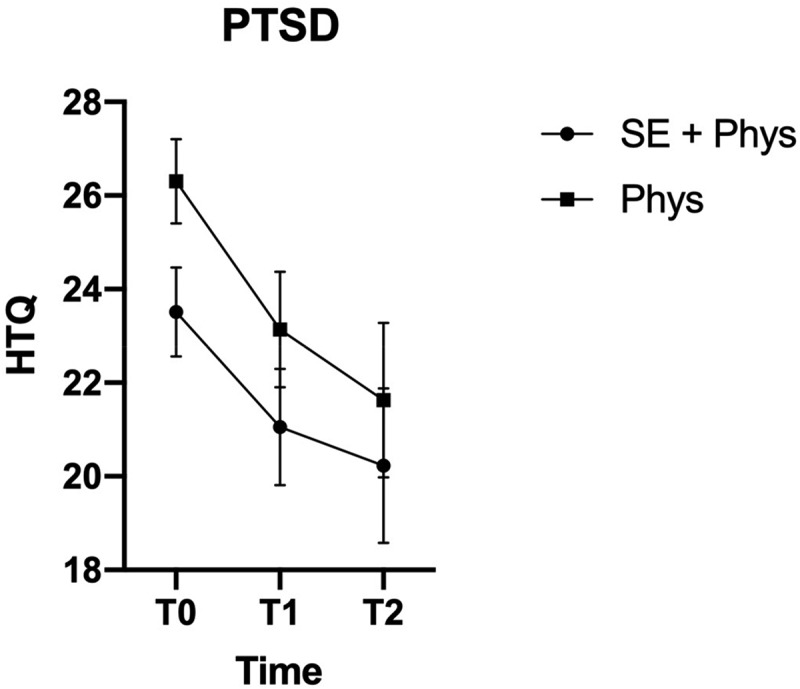
Marginal Means and Confidence Intervals for PTSD by Treatment Group and Time Point

Marginal means and 95% confidence intervals on the RMDQ (Roland morris disability questionnaire). T0 = baseline before randomisation, T1 = 6-month follow up, T2 = 12-month follow up. SE+Phys = Somatic Experiencing® + Physiotherapy, Phys = physiotherapy.

In terms of effect sizes, the unadjusted mean differences for both groups, SE+PT and PT respectively, revealed a moderate to large effect on pain-related disability (RMDQ) from baseline to T1 (Cohen’s *d* = 0.75; 0.67). It corresponds to a 22–27% reduction in pain-related disability and is considered more than a minimal clinically important difference (MCID) in the chronic sample in the current study (Jordan, Dunn, Lewis, & Croft, 2006; Lauridsen et al., 2006; Patrick et al., 1995). For PTSS (HTQ), the improvement was only of small to medium size from baseline to T1 (Cohen’s *d* = 0.23; 0.41), which is not above the MCID.

## 3. Discussion

The aim of the current study was to assess whether an additional SE intervention in combination with a PT intervention for comorbid PTSS and LBP would reduce pain-related disability compared with PT alone. It was hypothesised that the additional intervention would have a larger effect on pain-related disability at the 6-month follow up, as well as on all the secondary outcomes.

Contrary to expectations, there were no significant group differences on any of the outcomes at any timepoints. Both groups achieved a significant reduction in pain-related disability at the 6 – and 12-month follow up, corresponding to 20–27% reduction in pain-related disability compared with baseline as measured on the RMDQ. The reduction in pain-related disability was slightly larger than what was achieved in the SE trial by Andersen et al. (2017). Also, both groups achieved a small but significant reduction in PTSS. However, this difference was very small and is not above the MCID. This is opposite to that reported by Andersen et al. (2017), where only the SE group achieved a significant reduction in PTSS.

Since self-report questionnaires are found to be over-inclusive compared with diagnostic interviews assessing PTSD (Siqveland, Hussain, Lindstrøm, Ruud, & Hauff, 2017), it is highly likely that a substantial group of the included patients did not fulfill the diagnostic criteria for PTSD. For this reason, it is difficult to conclude whether patients with severe PTSD would have benefitted from the PT intervention alone. It is possible that patients fulfiling the full diagnostic criteria for PTSD would need an additional intervention also targeting comorbid PTSD. On one hand, this is to be expected from the number of studies finding PTSD to be a risk factor for poor recovery in musculoskeletal pain conditions (Moeller-Bertram et al., 2012). On the other hand, a small number of studies also indicate that interventions targeting mutually maintaining mechanisms such as fear-avoidance behaviours and pain-catastrophising (Andersen et al., 2016; Robinson et al., 2013; Sullivan et al., 2017), in themselves, may have a positive impact on both pain-related disability and PTSD symptomatology. But again, neither of these studies were on patients with a PTSD diagnosis validated by a diagnostic interview. Unfortunately, in the current study, effects were only measured after both interventions were completed. For this reason, we do not know whether the SE intervention alone would have had the same effect as the PT intervention alone.

Although PTSS are prevalent in chronic pain conditions, patients fulfiling all the diagnostic criteria for PTSD are less prevalent (Siqveland et al., 2017). This is a major methodological challenge when applying an RCT design, where adequate statistical power, when using those diagnostic criteria, would require a large number of patients. A future study should aim to include only patients who fulfil the complete diagnostic criteria for PTSD. This should be ensured by a structured diagnostic interview for PTSD. Since this is time-consuming and significantly reduces the number of patients fulfiling the inclusion criteria, it is recommended to apply another design which requires a smaller sample size such as experiential sampling or a N-of-1 trial design. An additional advantage of these designs is that multiple repeated measures allow for a more in-depth analysis of potential causal mechanisms, such as pain-catastrophising or fear-avoidance beliefs that may change during the intervention and thereby affect PTSS.

### 3.1. Limitations

The current study has a number of limitations. The most important limitation is the lack of a diagnostic interview assessing PTSD. Hence, patients without a diagnosis of PTSD may have been included in the study. Although the SE, as an intervention, is not limited to treatment of patients with a diagnosis of PTSD, the inclusion of patients with more general distress may have diluted the results. It is likely that the PT intervention alone was sufficient for more general distress symptoms but not for patients meeting the complete diagnostic criteria for PTSD. Finally, the design does not allow for an assessment of the effect of SE alone compared with the PT intervention. Also, with no waitlist control group, it is not possible to say whether the effect was due to any of the interventions. For these reasons, it is not possible to draw any definite conclusions about the effect of SE for patients with LBP and comorbid PTSD. However, trajectory studies have shown that the subgroup of patients with comorbid high levels of PTSS and pain tend to follow a trajectory characterised by little or no recovery in PTSS and pain over time (Andersen et al., 2016; Ravn, Karstoft, Sterling, & Andersen, 2018). Hence, this subgroup of patients in the current study would most likely not have recovered without any intervention, although such a conclusion would require the inclusion of a waitlist control group.

## 4. Conclusions

The Somatic Experiencing® intervention did not have any additional effect on any of the outcomes at any timepoint compared with the Physiotherapeutic intervention alone. However, the overall effect sizes were significant. In particular, the overall reduction in pain-related disability from baseline to the 6-month follow up was moderate to large and above the MCID. Compared with the earlier SE study (Andersen et al., 2017), where a more limited PT intervention was applied, the current study showed significantly larger effect sizes. Although large effects sizes were achieved for both groups, the PT intervention alone was as effective as the combined intervention.
